# Impact of the COVID-19 pandemic on patients suffering from musculoskeletal tumours

**DOI:** 10.1007/s00264-020-04636-4

**Published:** 2020-05-26

**Authors:** Martin Thaler, Ismail Khosravi, Andreas Leithner, Panayiotis J. Papagelopoulos, Pietro Ruggieri

**Affiliations:** 1 Department of Orthopaedic Surgery, Medical University of Innsbruck, Anichstr. 35, 6020 Innsbruck, Austria; 2 Department of Orthopaedics and Trauma, Medical University of Graz, Auenbruggerplatz 5, 8036 Graz, Austria; 3 First Department of Orthopedic Surgery, National and Kapodistrian University of Athens, Attikon University General Hospital, 1 Rimini Str, P.C. 12462, Haidari, Athens, Greece; 4 Department of Orthopaedics and Orthopaedic Oncology, University of Padova, Via Nicolò Giustiniani, 3, 35128 Padova, Italy

**Keywords:** Musculoskeletal tumor, Oncology, COVID-19, Healthcare, Pandemic, Sarcoma, Severe acute respiratory syndrome coronavirus 2, SARS-CoV-2

## Abstract

**Background:**

The aim of the current study was to evaluate the impact of the coronavirus disease (COVID-19) pandemic on musculoskeletal tumor service by conducting an online survey of physicians.

**Methods:**

The survey was conducted among the members of the ISOLS (International Society of Limb Salvage) and the EMSOS (European Musculo-Skeletal Oncology Society). The survey consisted of 20 questions (single, multiple-response, ranked): origin and surgical experience of the participant (four questions), potential disruption of healthcare (12 questions), and influence of the COVID-19 pandemic on the particular physician (four questions). A matrix with four different response options was created for the particular surgical procedures).

**Results:**

One hundred forty-nine physicians from five continents completed the survey. Of the respondents, 20.1% and 20.7% stated that surgery for life-threatening sarcomas were stopped or delayed, respectively. Even when the malignancy was expected to involve infiltration of a neurovascular bundle or fracture of a bone, still 13.8% and 14.7% of the respondents, respectively, stated that surgery was not performed. In cases of pending fractures of bone tumors, 37.5 to 46.2% of operations were canceled.

**Conclusion:**

The SARS-CoV-2 pandemic caused a significant reduction in healthcare (surgery, radiotherapy, chemotherapy) for malignancies of the musculoskeletal system. Delaying or stopping these treatments is life-threatening or can cause severe morbidity, pain, and loss of function. Although the coronavirus disease causes severe medical complications, serious collateral damage including death due to delayed or untreated sarcomas should be avoided.

**Electronic supplementary material:**

The online version of this article (10.1007/s00264-020-04636-4) contains supplementary material, which is available to authorized users.

## Introduction

The global medical community is currently facing new challenges due to the rapidly expanding coronavirus disease 2019 (COVID-19), which is caused by the severe acute respiratory syndrome coronavirus 2 (SARS-CoV-2). Its effects can be seen in social disruption, exceptional healthcare utilization, and economic instability worldwide. Many healthcare systems have continued to serve patients with urgent medical needs. However, the definition of what is to be classified as urgent disease differs. The need for intensive care units for COVID-19 patients exerts a strong influence on bedside procedures or operative intervention for non-COVID-19 patients.

Data on the disease’s impact on healthcare for non-COVID-19 patients is lacking. Some publications give recommendations and guidelines for physicians to ensure safety for themselves, their clinical staff, and their patients when they come into contact with patients who are suffering or have suffered from COVID-19 [1]. However, little attention in these dark days is paid to non-COVID-19 patients and their access to healthcare. The impression is that the entire medical community and/or political leaders are focusing on COVID-19 patients and other life-threatening diseases do not seem as important. Bone and soft tissue tumours are neoplasms that can cause significant morbidity and mortality. Therefore, urgent and ideal treatment in the form of an interdisciplinary approach should be provided for these musculoskeletal oncology patients [2]. Postponing, delaying, or stopping therapy can be followed by life-threatening consequences. Bone and soft tissue sarcomas cause significant morbidity and mortality [2], which lead to death if untreated.

Thus, the aim of the present surveys was to investigate a possible impact on investigating and treating musculoskeletal oncology patients by conducting an online survey. The survey was conducted among members of the ISOLS (International Society of Limb Salvage) and the EMSOS (European Musculo-Skeletal Oncology Society) in order to evaluate the situation of physicians tending to musculoskeletal oncology pathologies worldwide and their patients.

## Methods

As no patient data were involved, approval by an institutional review board was deemed unnecessary. The survey was conducted from April 6 to April 21, 2020.

The questionnaire contained 20 questions covering four different topics, namely four questions on the origin and surgical experience of the participant, 12 questions on potential cutbacks in orthopaedic healthcare, and four questions concerning the influence of the pandemic with the focus on surgeons. In terms of question types, 12 questions offered multiple response options and six permitted one option only. A matrix with four different response options was created for the particular surgical procedures and permitted only one response per procedure: yes: still performed; stopped; delayed; and not provided at our department (see Appendix 1 for survey details).

To collect the data, SurveyMonkey (http://www.surveymonkey.com), an online data collection program, was used.

All data gathered from the online database were calculated as frequencies and percentages.

## Results

The majority of the respondents (*n* = 152) were musculoskeletal oncology surgeons (91.1%), followed by paediatric physicians specialized in musculoskeletal oncology (9.6%). Most applicants worked in an academic medical centre (64.5%), while 20.5% and 15% worked in a public or private hospital, respectively. On average, the respondents had been in medical practice for 18.4 years (min 1 year, max 45 years). Geographically, physicians from Asia, Europe, Australia, North, and South America responded. Most respondents were from the USA (20), followed by Japan (14), India (13), and Italy (12).

Only 5.4% of participants stated that no changes had been made at their department, while 48% and 52% stated that all elective inpatient and outpatient surgery was stopped at their department, respectively. Elective inpatient (41.9%) and outpatient (35.8%) surgery was restricted, and 4.7% stated that all surgery had been stopped. A reduction in the surgeon’s surgical volume was reported by 85.8% of the respondents, as well as assignment to remote work from home (35.1%), to patient care outside their specialty (23%), and to more administrative work (42.6%). Moreover, 31.3% of the respondents were effectively not working due to deferral of elective surgery.

**Fig. 1:**
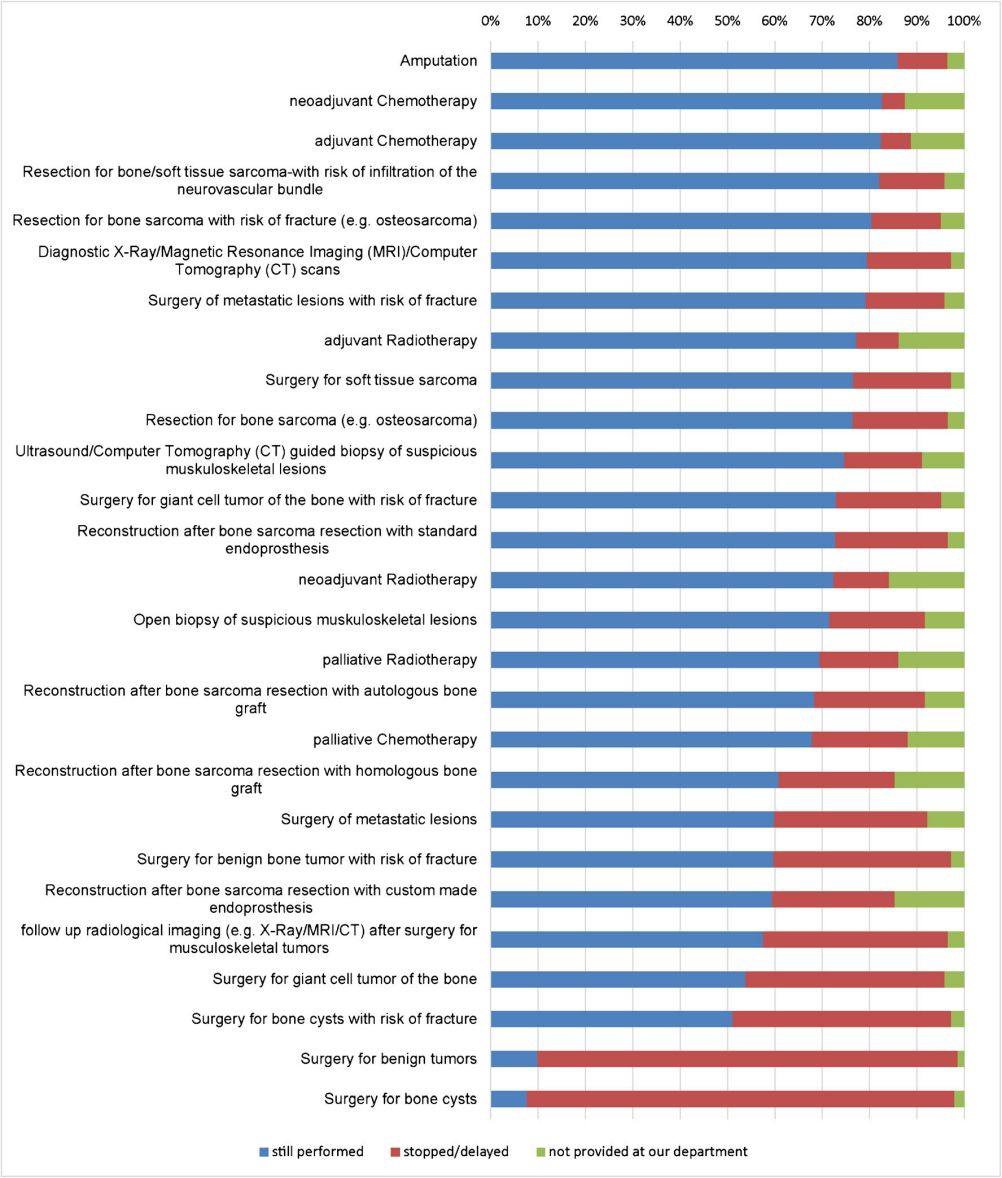
Participants’ responses whether dedicated procedures or surgery are currently being performed at their department. The response possibilities were as follows: yes, stopped or delayed, not provided at my department

Figure 1 and Table 1 show the impact of the COVID-19 pandemic on procedures and investigations in musculoskeletal oncology departments. Of the respondents 20.1% reported a stop or delay in surgery of bone sarcomas, 20.7% in surgery of soft tissue sarcomas, plus 20.1% in open and 16.4% in ultrasound/computed tomography (CT)-guided biopsy of suspicious musculoskeletal lesions. No resection for bone sarcoma with risk of fracture, and no resection of sarcoma with risk of infiltration of the neurovascular bundle was reported by 14.7% and 13.8% of the respondents, respectively. A stop or delay in palliative radiotherapy was reported by 16.7% and in palliative chemotherapy by 20.3% or the respondents, while 88.7% and 90.2% of the respondents reported a discontinuation of surgery for benign tumours and bone cysts, respectively. However, 85.9% of the respondents reported that amputations were still being performed.

**Table 1:** Percentage of dedicated procedures and investigations for musculoskeletal oncology patients evaluated by the respondents

	Still performed	Stopped/delayed	Not provided at our department
Amputation	85.9%	10.6%	3.5%
Neoadjuvant chemotherapy	82.6%	4.9%	12.5%
Adjuvant chemotherapy	82.4%	6.3%	11.3%
Resection for bone/soft tissue sarcoma-with risk of infiltration of the neurovascular bundle	82.1%	13.8%	4.1%
Resection for bone sarcoma with risk of fracture (e.g. osteosarcoma)	80.4%	14.7%	4.9%
Diagnostic X-ray/magnetic resonance imaging (MRI)/computed tomography (CT) scans	79.5%	17.8%	2.7%
Surgery of metastatic lesions with risk of fracture	79.2%	16.7%	4.2%
Adjuvant radiotherapy	77.2%	9.0%	13.8%
Surgery for soft tissue sarcoma	76.6%	20.7%	2.8%
Resection for bone sarcoma (e.g., osteosarcoma)	76.4%	20.1%	3.5%
Ultrasound/computed tomography (CT)-guided biopsy of suspicious musculoskeletal lesions	74.7%	16.4%	8.9%
Surgery for giant cell tumor of the bone with risk of fracture	72.9%	22.2%	4.9%
Reconstruction after bone sarcoma resection with standard endoprosthesis	72.7%	23.8%	3.5%
Neoadjuvant radiotherapy	72.4%	11.7%	15.9%
Open biopsy of suspicious musculoskeletal lesions	71.5%	20.1%	8.3%
Palliative radiotherapy	69.4%	16.7%	13.9%
Reconstruction after bone sarcoma resection with autologous bone graft	68.3%	23.5%	8.3%
Palliative chemotherapy	67.8%	20.3%	11.9%
Reconstruction after bone sarcoma resection with homologous bone graft	60.8%	24.5%	14.7%
Surgery of metastatic lesions	59.9%	32.4%	7.8%
Surgery for benign bone tumor with risk of fracture	59.7%	37.5%	2.8%
Reconstruction after bone sarcoma resection with custom made endoprosthesis	59.4%	25.9%	14.7%
Follow-up radiological imaging (e.g., X-ray/MRI/CT) after surgery for musculoskeletal tumors	57.5%	39.0%	3.4%
Surgery for giant cell tumor of the bone	53.9%	42.0%	4.2%
Surgery for bone cysts with risk of fracture	51.1%	46.2%	2.8%
Surgery for benign tumors	9.9%	88.7%	1.4%
Surgery for bone cysts	7.7%	90.2%	2.1%

**Fig. 2:**
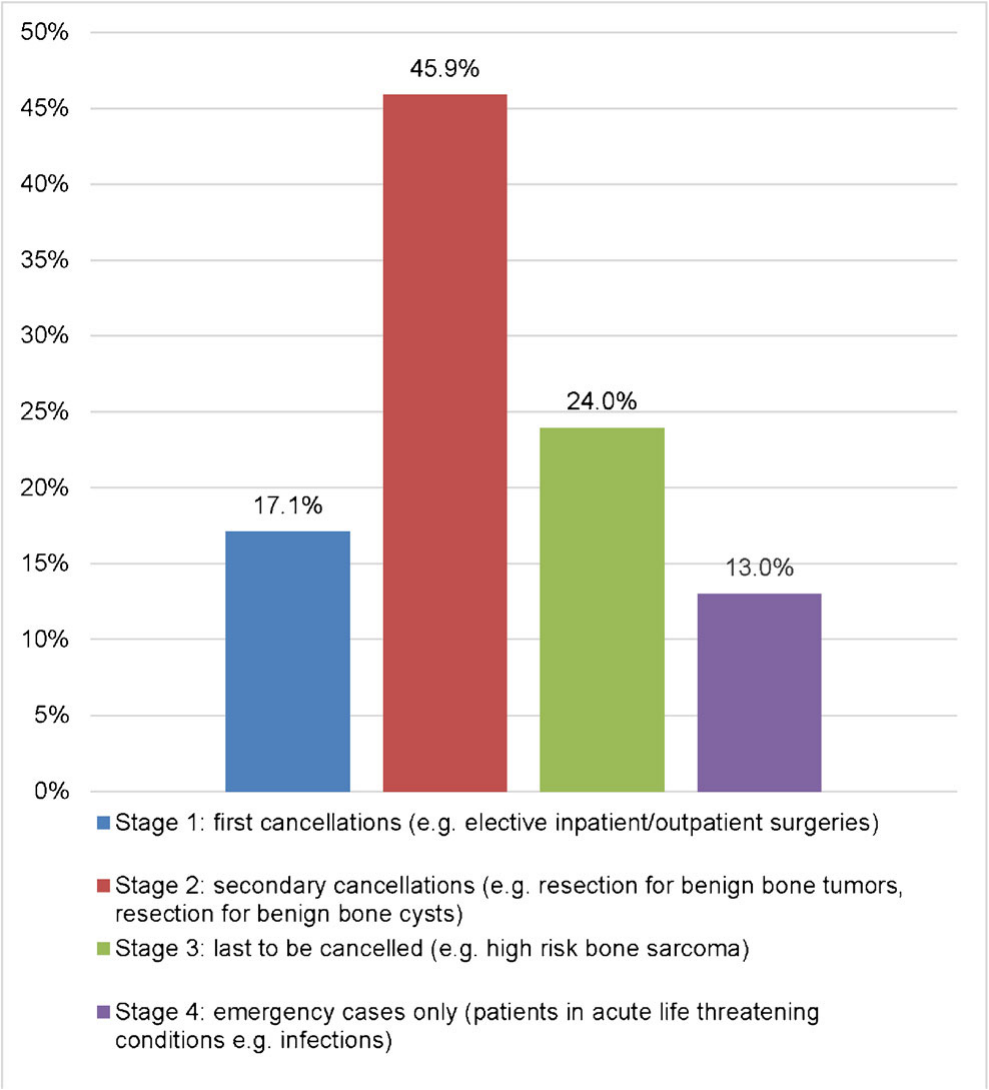
Participant’s responses showing the stages of escalation during the COVID-19 pandemic in their hospital

**Fig. 3:**
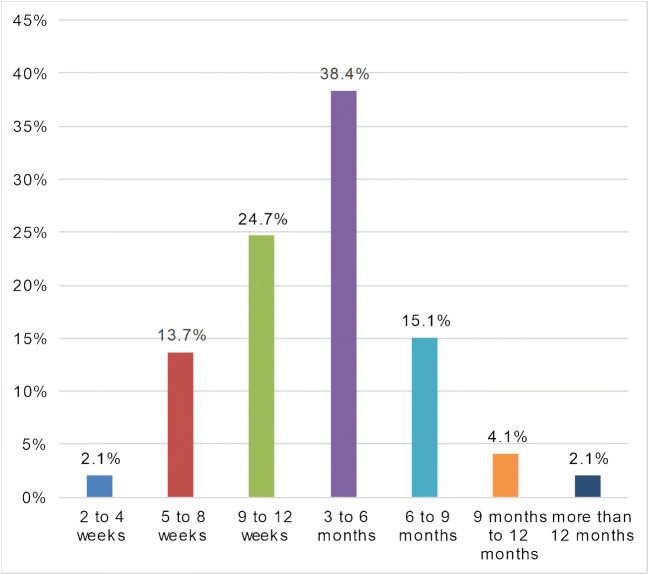
Participants’ responses on how long they think the COVID-19 pandemic will affect their clinical and surgical schedule

The stages of escalating-down activities at musculoskeletal oncology departments are presented in Fig. 2. The estimated time effect of the COVID-19 pandemic on musculoskeletal oncology is shown in Fig. 3.

Supply disruptions (74.3%) and staff disruptions (63.6%) were the most relevant constraints reported during the COVID-19 pandemic. Of all participants, 70.3% had received specific COVID-19 training, while only 15% of all respondents were not aware of a positive COVID-19 test at their healthcare unit (patient, medical and non-medical staff). Most meetings in hospitals were conducted exclusively online via videoconference (39.7%). Healthcare with the help of online videoconferences and telephone was reported by 52% and 68.9% of the respondents, respectively.

The results of the multiple-response question regarding the respondents’ fear of infecting family and friends are presented in Table 2. Table 3 shows the effect of the COVID-19 pandemic on the outpatient clinic as evaluated by the respondents.

**Table 2:** Evaluation of the fear of infecting family and friends and the preventive approach taken (multiple responses possible)

Do you fear that you could infect your family or friends and what is your preventive approach?	
Yes, I wash and disinfect my hands more often than usual	85.62%
Yes, I am more careful at work than usual	84.25%
Yes, I change my clothes at the hospital more often	41.10%
Yes, I disinfect surfaces in my home after I touch them	28.77%
Yes, I avoid close physical contact with my family members	25.34%
Yes, I try to keep a distance from my family at home	21.92%
Other (please specify)	10.96%
Yes, I wear a surgical mask/other protection at home	10.27%
Yes, I took off from work	9.59%
Yes, I do not stay in the same room with other members of my family	6.85%
Yes, I do not go home anymore (stay at the hospital, hotel, second apartment, etc.)	4.11%
No, I do not care at all	1.37%
No, I have not thought about this situation	0.00%

**Table 3:** Effect of the COVID-19 pandemic on the outpatient clinic as evaluated by the respondents

What specific effect has the COVID-19 pandemic had on your outpatient clinic?	
ALL patients are being tested for SARS-CoV-2 prior to orthopedic clinical examination	2.1%
No changes at our outpatient clinic	4.8%
Other (please specify)	8.9%
Patients with positive symptoms/positive screening questions are being tested for SARS-CoV-2	17.8%
Only patients with acute symptoms (fracture, infection, tumor e.g. bone sarcoma) are allowed at our outpatient clinic	29.5%
ALL patients are screened for symptoms and fill out a questionnaire before clinical examination	37.0%

Of the participants, 50.3% reported that follow-up investigations were performed clinically and 51% radiologically, while 20.4% of the participants stated that patients were no longer followed up after treatment. With regard to physical therapy and rehabilitation, 43.8% of the participants stated that these were available for selected cases and 19.9% that these were not available.

## Discussion

The recent COVID-19 outbreak is deemed a global health emergency. Internationally, the number of confirmed infections, deaths, and sick persons has continued to rise, and the global impact of this viral infection is one of heightening concern. Many countries decided to institute a lockdown and quarantine their citizens. The potential effect of this virus on the world’s healthcare systems is unpredictable and many nations have decided to focus their resources on COVID-19 patients. However, non-COVID-19 patients are also waiting for their investigations, treatments and follow-up investigations. At the beginning of 2020, it is hard to predict the consequences that the cutback and redirection of healthcare resources will have on our patients during the COVID-19 pandemic.

A general reduction in the number of cases treated at musculoskeletal oncology departments was found in the current study. Thus, the reduction in case numbers severely affected the daily practice of participating physicians, with a reduction in surgical volume and severe effects on elective inpatient and outpatient surgeries. The majority (62.1%) of the respondents stated that they expected the pandemic to affect clinical routine and surgical schedule for nine  weeks to six months (Fig. 3).

Already, scattered reported COVID-19 pandemic cutbacks on healthcare have caused a 25% reduction in major organ transplantations in Italy in four weeks due to the limited number of intensive care units [3]. Furthermore, Connor et al. [4] expressed their concerns about the implications of the pandemic with regard to urology patients, especially those with malignant disease. The results of our study show drastic impairment of musculoskeletal oncology services worldwide. Resection of bone and soft tissue sarcomas, which untreated lead to patient death [5], were reported by approximately 20% of the respondents to be discontinued in their department. Bone sarcomas and soft tissue sarcomas can infiltrate the neurovascular bundle or cause a severe pathological fracture, which can compromise the oncologic resection. Consequently, limb salvage might no longer be possible and patients would have to be scheduled for ablative final operations [6, 7].

Hence, surgery for these kinds of tumour is mandatory at an early stage in order to prevent extensive morbidity for the patient [8]. Even in these scenarios, 13.8 to 14.7% of the participants reported that surgery was not performed at their musculoskeletal oncology department. The only reason for stopping or delaying this fundamental treatment for patients undergoing surgery for bone or soft tissue sarcoma would have been the unavailability of the required intermediate or intensive care units. Accordingly, the reallocation of intermediate and intensive care units to COVID-19 patients would be the only reason for the fundamental reduction of this vital therapy for sarcoma patients. Amputations were still reported by a high rate of participants (85.9%), giving rise to the assumption that due to the COVID-19 pandemic, some patients, who might have been candidates for a limb salvage procedure, underwent an ablative procedure in order to save their life. In addition, a stop or delay in reconstruction after sarcoma resection was reported by 23.5% (autologous bone graft) to 25.9% (custom made endoprosthesis) of the participants (Fig. 1, Table 1). Another reason for this scenario might have been to reduce contamination (staff, patients, community) in an “open environment” such as hospitals, which are difficult to control, in order to prevent contamination of immunocompromised patients (cancer patients).

Soft tissue sarcomas and bone sarcomas are rare entities, accounting for approximately 1% and 0.2% of all malignancies, respectively [9]. However, the relative incidence of bone metastasis by type of tumour, in patients with advanced metastatic disease, is 65–75% in breast carcinoma, 65–75% in prostate carcinoma, 60% in thyroid carcinoma, 30–40% in lung carcinoma, 40% in bladder carcinoma, 20–25% in renal cell carcinoma, and 14–45% in melanoma [10]. Although bone metastasis is a major cause of morbidity characterized by severe pain, impaired mobility, pathologic fractures, and spinal cord compression, only 59.7% of our respondents reported that patients were receiving surgical treatment for this pathology during the COVID-19 crisis.

The delay in taking biopsies of musculoskeletal tumors and consequently the delay in diagnosis results in a lesion that at surgery is larger in size, which might compromise oncologic outcome. In addition, the delay produces increased cost, pain, and patient anxiety [11, 12]. Although these facts are well known, 20.1% and 16.4% of the participants stated that open and ultrasound/CT-guided biopsies of suspicious lesions had been stopped and delayed, respectively. Under normal circumstances, none of the biopsy patients would require an intermediate or intensive care unit.

The majority of respondents were still administering neoadjuvant (82.6%) and adjuvant chemotherapy (82.4%), as well as neoadjuvant (72.4%) and adjuvant radiotherapy (77.2%). Although palliative chemotherapy and radiation therapy can relieve symptoms and improve quality of life in seriously ill patients [13], these therapies were reported by 16.7% and 20.3% of our respondents to have been stopped or delayed, respectively.

Benign bone tumours and bone cysts are the most common lesion in the immature skeleton and mostly appear in the proximal femur and humerus [14]. Although these lesions tend to fracture, which would require surgery to avoid malalignment of the bone and to reduce the morbidity of pain and fracture [15], only 51.1% of our participants stated that these patients were being treated at their department.

However, not only non-COVID-19 but also seriously ill patients with life-threatening diseases are suffering under the pandemic, just as are the physicians’ work and social life, as shown in our study. Although some publications deal with anxiety, depression, indignation, happiness, and cognitive indicators caused by the COVID-19 pandemic [16], no publications report the effect of the pandemic on the social life of physicians. Our study found that the pandemic is having an enormous impact on physicians’ conduct toward their families at home. Only 1.3% of the participants reported not having made any changes in their home behaviour at all. However, 68.25% reported isolating themselves in various ways, namely 21.6% kept a distance from other family members, 25.7% avoided any physical contact with them, 6.8% reported living in separate rooms at home, and 4% were no longer going home until further notice, as they feared they could potentially harm their family (Table 2). Aware of the risk of healthcare professionals such as physicians contracting COVID-19, musculoskeletal physicians are understandably trying to protect the ones closest to them by practicing some form of self-imposed social distancing in their private life.

Several limitations of the study have to be acknowledged. First, the findings presented in our study are an expert opinion and therefore represent a low level of evidence (V). However, EMSOS and ISOLS members are internationally well-known specialists and can therefore be assumed to be opinion leaders in the field of musculoskeletal oncology. Moreover, in most countries, the treatment of sarcomas and musculoskeletal tumors is centralized to highly specialized sarcoma centres staffed by ISOLS and EMSOS members, namely experts and opinion leaders in their specialization. Second, our respondents come from various countries that at the time of the survey maintained different strategies toward the pandemic and were experiencing the pandemic at different levels of severity.

## Conclusion

So far, the harm for musculoskeletal patients as a result of this unique situation is difficult to evaluate with the acquired data, which present only an initial picture. However, this massive reduction in musculoskeletal oncology services might have drastic consequences for affected patients. Meanwhile, every effort should be undertaken to ensure that all delayed or postponed sarcoma patients acquire safe access to treatment, even during the COVID-19 pandemic. Still, COVID-19 will continue to affect the healthcare system, hospitals, practices, surgeons, and patients for the foreseeable future. Although the coronavirus disease causes severe medical complications and leads to death in some people, we must continue to care for other seriously ill patients, who need urgent and dedicated treatment.

## Electronic supplementary material

ESM 1 (PDF 666 kb)
