# Supplementary material for: Impact of the COVID-19 pandemic on patients suffering from musculoskeletal tumours
Source: Int Orthop. 2020 May 26;44(8):1503–9. doi: 10.1007/s00264-020-04636-4 (PMC7247966; doi:10.1007/s00264-020-04636-4)
Supplement: Supplementary file 1 — (PDF 666 kb) [file 264_2020_4636_MOESM1_ESM.pdf]

# COVID-19 Musculoskeletal Oncology Survey

Thank you for participating in this survey.

We would like to ask you to kindly take approximately 8 minutes of your time andcomplete an online survey regarding the COVID-19 pandemic and how it affects yourpatients and your practice.

All data and questions will be absolutely anonymous. All answered questions will be saved so that you will be able to return to your latest question and go on with the survey fromthat question.

1. In which country are you currently working?

Country ______

2. What is your orthopedic speciality? (Mark all that apply)

- ☐ Orthopedics - musculoskeletal oncology
- ☐ Orthopedics - general
- ☐ Radiology - oncology
- ☐ Radiotherapy
- ☐ Pathology
- ☐ Internal medicine - oncology
- ☐ Internal medicine - general
- ☐ Pediatrics - musculoskeletal oncology
- ☐ Pediatrics - general
- ☐ Pediatric surgery
- ☐ Plastic surgery
- ☐ Resident/ Fellow - not specified yet
- ☐ None of the above or other (please specify)

______

3. In which type of environment are you working? (Mark all that apply)

- ☐ Academic medical center
- ☐ Public hospital
- ☐ Private hospital

4. How many years have you been practicing?

______

5. Do you fear infecting your friends or family and what is your approach forprevention? (Mark all that apply)

- [ ] Yes, I don't go home anymore (stay at the hospital, hotel, second apartement, etc.)
- [ ] Yes, I wash and disinfect my hands more often than usual
- [ ] Yes, I change my clothes in the hospital more often
- [ ] Yes, I try to keep a distance to my family at home
- [ ] Yes, I don't stay in the same room with other members of my family anymore
- [ ] Yes, I disinfect surfaces in my home after I touch them
- [ ] Other (please specify)
- [ ] Yes, I wear a surgical mask/other protection at home
- [ ] Yes, I avoid close physical contact with my family members
- [ ] Yes, I am more careful at work than usual
- [ ] Yes, I took off from work
- [ ] No, I don't care at all
- [ ] No, I haven't thought about this situation

6. What specific effects has the COVID-19 pandemic had on your department?(Mark all that apply)

- [ ] No changes at the department
- [ ] All surgeries have been stoppedstopped
- [ ] The department has **stopped** *elective inpatient* surgery
- [ ] The department has **stopped** elective *outpatient* surgery
- [ ] The hospital has **selectively restricted** *inpatient* elective surgery
- [ ] The hospital has **selectively restricted** *outpatient* elective surgery

7. What specific effect has the COVID-19 pandemic had on your outpatientclinic?

- ( ) **ALL** patients are being tested for SARS-CoV-2 prior to orthopedic clinical examination
- ( ) **ALL** patients are being screened for SARS-CoV-2 (e.g. having bodytemperature taken, answering a questionnaire, etc.) prior to orthopedicclinical examination
- ( ) Patients with positivesymptoms/positive screening questionsare being tested for SARS-CoV-2
- ( ) Other (please specify)
- ( ) No changes at our outpatient clinic
- ( ) Only patients with acute orthopedicsymptoms (fracture, infection, tumor eg.bone sarcoma) are allowed at ouroutpatient clinic

8. How has the COVID-19 pandemic affected YOUR practice as an orthopedic surgeon? (Mark all that apply)

- [ ] No impact
- [ ] My volume of performed surgery is reduced
- [ ] Discussing the delay due to the pandemic with patients
- [ ] No Training or teaching (students,residents, fellows) due to the pandemic
- [ ] More non-surgical orthopedic clinicalcare is being performed
- [ ] More administrative work is being done
- [ ] Orthopedic surgeons are assigned moreoften to non-orthopedic patient care due to the pandemic

9. Are the following procedures currently being performed currently being performed at your departement?

| | still performed | stopped | delayed | not provided at our department |
|---|---|---|---|---|
| Ultrasound/ Computer Tomography (CT) guided biopsy of suspicious muskuloskeletal lesions | ○ | ○ | ○ | ○ |
| Open biopsy of suspicious muskuloskeletal lesions | ○ | ○ | ○ | ○ |
| Diagnostic X-Ray/Magnetic Resonance Imaging (MRI)/ Computer Tomography (CT) scans | ○ | ○ | ○ | ○ |
| follow up radiological imaging (e.g. X-Ray/MRI/CT) after surgery for musculoskeletal tumors | ○ | ○ | ○ | ○ |
| Surgery for soft tissue sarcoma | ○ | ○ | ○ | ○ |
| Resection for bone sarcoma (e.g. osteosarcoma) | ○ | ○ | ○ | ○ |
| Resection for bone sarcoma with risk of fracture (e.g. osteosarcoma) | ○ | ○ | ○ | ○ |
| Resection for bone/soft tissue sarcoma with risk of infiltration of the neurovascular bundle | ○ | ○ | ○ | ○ |
| Reconstruction after bone sarcoma resection with standard endoprosthesis | ○ | ○ | ○ | ○ |
| Reconstruction after bone sarcoma resection with custom made endoprosthesis | ○ | ○ | ○ | ○ |
| Reconstruction after bone sarcoma resection with homologous bone graft | ○ | ○ | ○ | ○ |
| Reconstruction after bone sarcoma resection with autologous bone graft | ○ | ○ | ○ | ○ |
| Surgery of metastatic lesions | ○ | ○ | ○ | ○ |
| Surgery of metastatic lesions with risk of fracture | ○ | ○ | ○ | ○ |
| Surgery for benign tumors | ○ | ○ | ○ | ○ |
| Surgery for benign bone tumor with risk of fracture | ○ | ○ | ○ | ○ |
| Surgery for bone cysts | ○ | ○ | ○ | ○ |

| | | | | |
|---|---|---|---|---|
| Surgery for bone cysts with risk of fracture | ◯ | ◯ | ◯ | ◯ |
| Surgery for giant cell tumor of the bone | ◯ | ◯ | ◯ | ◯ |
| Surgery for giant cell tumor of the bone with risk of fracture | ◯ | ◯ | ◯ | ◯ |
| adjuvant Chemotherapy | ◯ | ◯ | ◯ | ◯ |
| neoadjuvant Chemotherapy | ◯ | ◯ | ◯ | ◯ |
| palliative Chemotherapy | ◯ | ◯ | ◯ | ◯ |
| adjuvant Radiotherapy | ◯ | ◯ | ◯ | ◯ |
| neoadjuvant Radiotherapy | ◯ | ◯ | ◯ | ◯ |
| palliative Radiotherapy | ◯ | ◯ | ◯ | ◯ |
| Amputation | ◯ | ◯ | ◯ | ◯ |

10. Imagine 4 Stages of escalating down activities, in *which stage* is your department department *at this time*?

◯ *Stage 1:* first cancellations (e.g. elective inpatient/outpatient surgeries)

◯ *Stage 2:* secondary cancellations (e.g. resection for benign bone tumors, resection for benign bone cysts)

◯ *Stage 3:* last to be cancelled (e.g. high risk bone sarcoma)

◯ *Stage 4*: emergency cases only (patients in acute life threatening conditions e.g. infections)

11. Has there been any specific COVID-19 training for your surgical staff?

◯ yes

◯ no

12. Has there been a positive COVID-19 test result (infection proved)? (Mark allthat apply)

☐ Patient in my hospital

☐ Patient in my department

☐ Health care professional in my hospital

☐ Health care professional in my department

☐ Other staff in my hospital

☐ Other staff in my department

☐ None of the above

13. Have there been any disruptions related to the pandemic? (Mark all thatapply)

- [ ] Staff disruptions
- [ ] Supply disruptions
- [ ] Missing regular intensive care units
- [ ] Missing regular intermediate care units
- [ ] Missing regular inpatient beds
- [ ] Missing COVID-19 intensive care units
- [ ] Missing COVID-19 intermediate care units
- [ ] Missing COVID-19 inpatient beds
- [ ] Other (please specify)

14. With regard to your regular meetings, is there any difference due to theCOVID-19 pandemic?

- ( ) No difference at my department
- ( ) Reduced staff at the meetings
- ( ) No meetings anymore
- ( ) Other (please specify)
- ( ) **ALL** staff members participate at the meetings but keep a distance to eachother
- ( ) **ALL** staff members participate at the meetings but wear protection (masks,coats etc.)
- ( ) Meetings are held exclusively online via videoconference

15. What are your clinic's approaches to preventing sick leave due to COVID-19 pandemic? (Mark all that apply)

- [ ] Staff is separated into groups
- [ ] Reduced staff appearance at meetings
- [ ] Altered rotations in staff appearance
- [ ] Personal protection (masks, coats,gloves etc.)
- [ ] Remote working from home (scientificresearch, webinars, telemedicine etc.)
- [ ] No prevention

16. Do you offer patient care via technologies like telemedicine? (Mark all thatapply)

- [ ] Videoconference (Skype, Zoom, etc.)
- [ ] web based telemedicine (FaceTime, GoogleChat etc.)
- [ ] None
- [ ] Other (please specify)
- [ ] Telephone
- [ ] EHR/EMR (electronic healthrecord/ electronic medical record)

17. How long do you think the COVID-19 pandemic will affect your clinicalroutine/your surgical schedule?

- ( ) 2 to 4 weeks
- ( ) 5 to 8 weeks
- ( ) 9 to 12 weeks
- ( ) 3 to 6 months
- ( ) 6 to 9 months
- ( ) 9 to 12 months
- ( ) more than 12 months

18. What impact has the COVID-19 pandemic had on you? (Mark all that apply)

- [ ] I am effectively not involved in any surgical activity due to institutional or self-imposed deferral of elective surgery
- [ ] At this time, no confirmed cases of COVID-19 have occurred in my community or institution
- [ ] I am not working due to personal illness, COVID-19 exposure, or post-travel quarantine
- [ ] Confirmed COVID-19 cases have occurred in my community or institution
- [ ] I consider myself in a high-risk group for COVID-19 (age > 60, underlying condition e.g. high blood pressure, diabetes, cardiovascular disease)
- [ ] None
- [ ] Other (please specify)

19. Do you still perform follow up investigations on TJA patients? (Mark all that apply)

- [ ] Yes, clinical follow up
- [ ] Yes, but I follow up only high risk patients (e.g. complex revisions, septic, etc.)
- [ ] Yes, radiological follow up
- [ ] No, the sutures are taken out by someone else (e.g. general physician/family doctor)
- [ ] Yes, I take the sutures out myself
- [ ] No, the patients are not followed up anymore
- [ ] Other (please specify)

20. Is any physical therapy or rehabilitation offered for already discharged TJA patients? (Mark all that apply)

- [ ] Yes, inpatient
- [ ] No
- [ ] Yes, outpatient
- [ ] Only for selected cases
- [ ] Other (please specify)
